# Incidence of skin and soft tissue infections in ambulatory and inpatient settings, 2005–2010

**DOI:** 10.1186/s12879-015-1071-0

**Published:** 2015-08-21

**Authors:** Loren G. Miller, Debra F. Eisenberg, Honghu Liu, Chun-Lan Chang, Yan Wang, Rakesh Luthra, Anna Wallace, Christy Fang, Joseph Singer, Jose A. Suaya

**Affiliations:** Infectious Disease Clinical Outcomes Research Unit (ID-CORE), Division of Infectious Diseases, Los Angeles BioMedical Research Center at Harbor-UCLA, Torrance, CA USA; Division of Infectious Diseases, Harbor-UCLA Medical Center, 1000 W Carson St Box 466, Torrance, CA 90509 USA; David Geffen School of Medicine at UCLA, Los Angeles, USA; HealthCore, Inc, Wilmington, DE USA; Division of Public Health, School of Dentistry at UCLA, Los Angeles, USA; Division of General Internal Medicine, David Geffen School of Medicine at UCLA, Los Angeles, USA; Department of Biostatistics, Fielding School of Public Health at UCLA, Los Angeles, USA; U.S. Health Outcomes, GlaxoSmithKline, Research Triangle Park, Philadelphia, PA USA; U.S. Health Outcomes, NACMA, Vaccines, GlaxoSmithKline, Philadelphia, PA USA

## Abstract

**Background:**

The emergence of community-associated methicillin-resistant *S. aureus* was associated with dramatically increased skin and soft tissue infection (SSTI) incidence in the first few years of the 21^st^ century in the U.S. However, subsequent trends are poorly understood.

**Methods:**

We examined ambulatory and inpatient data of over 48 million persons years aged 0–64 years from the HealthCore Integrated Research Database (HIRD) between 2005 and 2010. Data were extracted from medical, pharmacy, and eligibility databases. We quantified SSTI incidence, type, and complications and comparative incidence trends for urinary tract infections (UTIs) and pneumonia.

**Results:**

A total of 2,301,803 SSTIs were identified. Most SSTIs (95 %) were treated in the ambulatory setting and most (60 %) were categorized as abscesses or cellulitis. During the study period, SSTI incidence remained relatively stable from 47.9 (95 % CI: 47.8–48.1) cases/1,000 PY in 2005 to 48.5 cases/1,000 PY (95 % CI: 48.3–48.6) in 2010). Persons aged 45–64 years had the highest incidence of both ambulatory-treated and inpatient-treated SSTIs (51.2 (95 % CI: 51.1–51.3) and 3.87 (95 % CI: 3.84–3.90) cases/1,000 PY, respectively). SSTI complications such as myositis, gangrene, and sepsis occurred in 0.93 % (95 % CI: 0.92–0.94 %) and 16.92 % (95 % CI: 16.87–16.97 %) of ambulatory-treated and inpatient-treated patients, respectively. SSTI incidence was approximately twice that of UTIs and tenfold of that of pneumonia.

**Conclusions:**

Among our large, diverse population of persons less than 65 years, SSTI incidence 2005 through 2010 has remained relatively constant at approximately 4.8 SSTIs per 100 person years, suggesting that previously observed increases in SSTI incidence remain sustained.

## Background

Skin and soft tissue infections (SSTIs) are a common reason to seek medical care in the United States (U.S.), and worldwide [1, 2]. SSTIs can lead to complications with significant morbidity, including hospitalization, surgical procedures, bacteremia, and death [3, 4].

Reports from the early 2000s suggest that SSTI incidence in the U.S. was rising. An investigation using data from the National Ambulatory Medical Care Survey and National Hospital Ambulatory Medical Care Survey found that visits for SSTIs to U.S. physician offices, hospital outpatient departments, and emergency departments increased by 50 % between 1997 and 2004 [1]. U.S. emergency department visits for SSTIs increased from an estimated 1.2 million visits annually in 1993 to 3.4 million in 2005 [2]. Data from the Healthcare Cost and Utilization Project National Inpatient Sample (HCUP) found that hospital admissions for SSTIs increased by 29 % during 2000–2004 [5]. There was no similar rise noted for other infectious diseases [5].

SSTIs constituted the most rapidly increasing reason for hospitalizations between 1997 and 2007, with stays for skin and subcutaneous tissue infections rising 90 % for men and 75 % for women [6]. Increases in SSTI incidence appear to be driven by the dramatic increase in community-associated methicillin-resistant *Staphylococcus aureus* (CA-MRSA) skin infections [1, 2, 5]. However, since this time there are few subsequent data on whether incidence of SSTIs in both ambulatory and inpatient settings has peaked, stabilized, or continued to rise. Additionally, there are no investigations that have taken a holistic approach to quantifying SSTI incidence in both the ambulatory and inpatient settings. To better understand trends in SSTI incidence, we reviewed administrative claims data within a large database of commercially insured members, who have full medical and pharmacy eligibility.

## Methods

We performed an observational retrospective cohort study utilizing administrative claims data. Data included medical and pharmacy claims from the HealthCore Integrated Research Database (HIRD). The HIRD data environment includes linked medical, pharmacy, and eligibility data from 14 health plans in the US, and includes health maintenance, point-of-service, preferred provider organizations and indemnity plans located in the northeastern, southeastern, mid-Atlantic, Midwestern, and western regions of the US. Data from the HIRD has been used previously in multiple incidence and cost studies [7–10] and includes information on over 48 million person years from 2005 to 2010.

For purposes of this analysis, we included all persons with at least one day of enrollment within the health plan. All data were handled in compliance with the Health Insurance Portability and Accountability Act (HIPAA) of 1996, and a limited dataset was used for the analyses. The limited dataset contained the fields of interest specific to the study including eligibility, medical and pharmacy information for the specified cohort during the study period and limits the amount of protected health information (PHI) in the dataset. Due to the fact that this research is observational in nature, with no intervention conducted on human subjects, and the research involves the analysis of existing administrative claims data, which is accessible by the investigators in such a manner that subjects cannot be identified, directly or through identifiers linked to the subjects, the study was approved as an exempt investigation by the Institutional Review Board at the Los Angeles Biomedical Research Institute at Harbor-UCLA.

SSTI incidence rates were obtained from the overall HIRD population. To calculate incidence, all SSTI episodes from 01/01/2005 through 12/31/2010 were included. Patient selection was defined as patients who had ≥1 medical (inpatient, emergency room [ER] or outpatient) claim with an ICD-9 diagnosis code for an SSTI between 01/01/2005 and 12/31/2010. The earliest occurrence was defined as the onset date for the first (index) SSTI episode. Any two consecutive claims for SSTIs or their complications within 30 days of each other were considered part of the same SSTI episode. A new SSTI episode therefore occurred if a SSTI claim was preceded by at least 31 days without any claim for SSTI or complication. Second, incidence rates were then calculated by dividing the number of all SSTIs during the study period by the person-years (PY) contributed by each member during the calendar year. To assess whether potentially observed trends in SSTI were related to changes in the HIRD population or our methodology, we assessed annual incidence of two common infections occurring in the ambulatory and inpatient settings, pneumonia and urinary tract infections (UTIs) from 2005 to 2010, for which we hypothesized there would be no significant changes in infection incidence.

ICD-9 codes for SSTIs (Table 1) were identified based on definitions from previous investigations utilizing administrative claims data for identification of SSTIs [1, 2, 4, 5]. If an SSTI episode had claims with multiple clinical diagnoses for SSTIs, a hierarchy was implemented to categorize the type of SSTI episode and the infection was categorized as the more severe infection. The following categorization hierarchy, based on clinical judgment, was used (starting with most severe): (1) infection due to device or graft; (2) surgical site infection; (3) non-healing surgical wound; (4) decubitus ulcer; (5) mastitis; (6) cellulitis/abscess; (7) erysipelas; (8) other skin and soft tissue infections; (9) furuncle/carbuncle; (10) impetigo; and (11) folliculitis. We also used ICD-9 codes to identify pneumonias (481.xx, 482.xx, 483.xx, 485.xx and 486.xx) and UTIs (590.xx and 599.0) employing the same rules for SSTI claims outlined above, in which claims within 30 days of each other were considered to be part of the same episode. ICD-9 codes were based on pneumonia and UTI definitions from previous investigations utilizing administrative claims data [11–13]. Because of limitations of the HIRD extraction process, we used data on pneumonia and UTIs from 2005 to 2009.

**Table 1:** Skin and soft tissue infections (SSTI) by clinical condition, severity, and ICD-9 code

Description	ICD-9 codes
Uncomplicated SSTI	
Carbuncle and furuncle	680.x
Cellulitis and abscess^a^	681.x-682.x
Erysipelas^a^	035.x
Impetigo	684.x
Mastitis	611.0x, 771.5x
Folliculitis^a^	704.8x
Complicated SSTI	
“Other” skin and subcutaneous tissue infections	686.x
Decubitis Ulcer^b^	707.x
Infection due to device or graft	996.6x
Surgical site infection	998.5x, 999.3x
Non-healing surgical wound^b^	998.83
Any SSTI diagnosis in a patient with any of the following comorbidities:	
Diabetes	250.xx
Chronic kidney disease	585.xx, 403.xx
Chronic liver disease	571.xx, 572.xx
Peripheral artery disease	440.xx, 443.9
Neuropathy	356.xx, 357.xx, 250.6x
Obesity	278.0x, 278.00, 278.01, 278.02, V85.21, V85.22, V85.23, V85.24, V85.25, V85.4, V85.3x
Alcohol or drug abuse	303.xx, 304.xx, 305.xx
Compromised immune function	279.0x, 279.1x, 279.2x, 279.3x, 334.8x

^a^Cellulitis, erysipelas, and folliculitis were considered complicated if the index diagnosis of the primary diagnosis was on an inpatient claim

^b^Decubitis ulcer and non-healing surgical wound were considered an active infection if: either a) clinical diagnosis was associated with concomitant prescription of any of the following of antibiotics: dicloxacillin, vancomycin, cefalexin, cefazolin, linezolid, daptomycin, clindamycin, trimethoprim-sulfamethoxazole, doxycycline, minocycline, quniupristin-dalfopristin within ± 10 days of the index diagnosis; or b) if no other concomitant clinical diagnosed infection occurred

Uncomplicated and complicated categories adapted from: Stevens DL et al. [15]

### Classification of SSTI episodes

#### Ambulatory-treatment vs. inpatient-treated of SSTI

SSTI episodes were classified as ambulatory-treated or inpatient-treated depending on where the first claim of the infection episode occurred. SSTIs whose first claim occured in the ambulatory care setting but resulted in a hospitalization were considered to be inpatient-treated. For ambulatory-treated infections, the proportion of episodes with a hospitalization was calculated. Further, any inpatient stay containing a clinical diagnosis of SSTI or its complications as one of the hospital discharge diagnoses during the infection episode was also captured. For inpatient-treated infections, we calculated re-hospitalization rates as the proportion of infections that had a subsequent hospitalization related to SSTI or its complications during the infection episode.

#### Complicated SSTIs vs. uncomplicated SSTIs

All SSTI episodes were categorized as either complicated or uncomplicated, based upon prior definitions [14, 15] in which complicated SSTIs were defined as a ICD-9 code diagnoses of severe SSTI or by the presence of a significant comorbid condition. Categories of severe SSTI diagnoses are detailed in Table 1. Comorbid conditions present during the pre-index period made any SSTI a complicated infection. These conditions included (a) chronic kidney disease; (b) chronic liver disease; (c) alcohol or drug abuse; (d) peripheral artery disease; (e) neuropathy; (f) diabetes; (g) obesity; and (h) compromised immune system (see Table 1 for ICD-9 codes).

#### Complications of SSTI episodes

Further, we examined relatively commonly described SSTI complications or sequelae [15]. These complications included lymphadenitis (ICD-9: 683.xx); myositis (ICD-9: 728.0x); necrotizing fasciitis (ICD-9: 728.86); gangrene (ICD-9: 040.0x, 785.4x); osteomyelitis (ICD-9:730.xx); bacteremia (ICD-9:790.7x); endocarditis (ICD-9:391.1x); and septicemia or sepsis (ICD-9: 038.x, 040.82, 785.52). An SSTI occurrence was considered to have a complication if the complication occurred during the infection episode. Based on clinical experience, osteomyelitis was flagged as associated with an SSTI if it was preceded by an SSTI claim within 90 days.

Statistical comparisons were made using the chi-squared test and Cochrane-Armitage test, as appropriate. All analyses were performed using SAS 9.2 (Cary, North Carolina). A p value of *p* < 0.05 was considered to be statistically significant.

## Results

There were a total of 2,301,803 SSTIs in both the ambulatory and inpatient settings between 2005 and 2010. The vast majority (95 %) of SSTIs were diagnosed in the outpatient setting (ambulatory-treated; 45.73, 95 % confidence interval (CI): 45.67–45.79 episodes per 1,000 PY) vs. 2.19 (95 % CI: 2.17–2.20) episodes per 1,000 PY that were inpatient-treated (Table 2).

**Table 2:** Incidence rate of skin and soft tissue infections (SSTIs) by initial location of treatment and age group from 2005 through 2010

Initial location of treatment	SSTI cases	Person- years (PY)^a^	SSTI cases / 1,000 PY	95 % Confidence interval	
				Lower	Upper
Ambulatory setting					
0–17 years	500,434	11,500,647	43.51	43.40	43.63
18–44 years	898,215	20,960,045	42.85	42.77	42.94
45–64 years	798,182	15,579,211	51.23	51.12	51.34
0–64 years	2,196,831	48,039,902	45.73	45.67	45.79
Inpatient setting					
0–17 years	10,444	11,500,647	0.91	0.89	0.93
18–44 years	34,299	20,960,045	1.64	1.62	1.65
45–64 years	60,229	15,579,211	3.87	3.84	3.90
0–64 years	104,972	48,039,902	2.19	2.17	2.20

^a^After discounting episode duration

The age group with the highest incidence of SSTIs was 45–64 years in both the ambulatory-treated (51.23 SSTIs per 1,000 PY, 95 % CI: 51.12–51.34) and the inpatient-treated SSTIs (3.87 SSTIs per 1,000 PY, 95 % CI: 3.84–3.90; Table 2). In the ambulatory-treated SSTIs, differences in SSTI incidence were relatively modest between this group and the younger age groups; SSTI incidence among those ages 0–17 years and 18–44 years was 43.51 (95 % CI: 43.40–43.63) and 42.85 (95 % CI: 42.77–42.94) SSTIs per 1,000 PY, respectively. However, among inpatient-treated SSTIs, incidence differences were more pronounced. Incidence was 0.91 (95 % CI: 0.89–0.93) and 1.64 (95 % CI: 1.62–1.65) SSTIs per 1,000 PY among those ages 0–17 and 18–44, respectively, compared to 3.87 (95 % CI: 3.84–3.90) SSTIs per 1,000 PY among those age 45–64 (*p* < 0.0001).

The clinical diagnosis of abscess or cellulitis was the most common SSTI, representing 57.32 % (95 % CI: 57.26–57.38 %) of SSTIs overall; 57.62 % (95 % CI: 59.90–60.03) in the ambulatory-treated and 51.13 % (95 % CI: 50.71–51.55) in the inpatient-treated groups (Table 3). The remaining cases of SSTIs (ambulatory and inpatient combined) were comprised of folliculitis (12.00 %, 95 % CI: 11.96–12.04 %)), impetigo (6.61 %, 95 % CI: 6.58–6.64 %)), furuncle (3.29 %, 95 % CI: 3.26–3.31 %)), mastitis (2.25 %, 95 % CI: 2.23–2.27 %)), surgical site infection (2.19 %, 95 % CI: 2.17–2.21 %)), decubitus ulcer (5.28 %, 95 % CI: 5.25–5.31 %)), device or graft (1.21 %, 95 % CI: 1.19–1.22 %)) and other (unspecified cases of SSTI; 9.25 %, 95 % CI: 9.21–9.29 %)). Distributions of SSTI type stratified by ambulatory-treated and inpatient-treated SSTIs are further outlined in Table 3.

**Table 3:** Complication rates associated with skin and soft tissue infections (SSTIs) by major initial clinical diagnosis and setting from 2005 through 2010

Initial location of treatment	Episodes (N, %)		Type of complication^ab^					
			Any complications	Lymphadenitis	Myositis/ Necrotizing fasciitis	Gangrene	Osteo-myelitis	Bacteremia/Endocarditis/Septicemia/ Sepsis
In ambulatory settings								
Abscess/Cellulitis^c^	1,265,736	57.62 %	0.93 %	0.14 %	0.03 %	0.08 %	0.46 %	0.31 %
Folliculitis	275,751	12.55 %	0.22 %	0.08 %	0.00 %	0.01 %	0.02 %	0.11 %
Impetigo	151,856	6.91 %	0.28 %	0.14 %	0.01 %	0.00 %	0.01 %	0.13 %
Furuncle	75,280	3.43 %	0.56 %	0.14 %	0.02 %	0.03 %	0.08 %	0.31 %
Mastitis	50,262	2.29 %	0.28 %	0.05 %	0.01 %	0.02 %	0.04 %	0.17 %
Surgical Site Infection	29,520	1.34 %	4.23 %	0.09 %	0.09 %	0.21 %	2.34 %	1.89 %
Decubitus Ulcer	111,320	5.07 %	6.77 %	0.02 %	0.13 %	1.47 %	5.06 %	1.67 %
Device or Graft	14,534	0.66 %	8.59 %	0.04 %	0.05 %	0.16 %	3.52 %	5.29 %
Non-healing Surgical Wound	12,159	0.55 %	4.69 %	0.05 %	0.26 %	0.87 %	2.32 %	1.71 %
Other	210,413	9.58 %	0.75 %	0.07 %	0.02 %	0.05 %	0.43 %	0.21 %
All	2,196,831	100.0 %	1.16 %	0.11 %	0.03 %	0.14 %	0.64 %	0.39 %
In inpatient settings								
Abscess/Cellulitis^c^	53,667	51.13 %	16.92 %	1.10 %	1.47 %	1.90 %	5.03 %	10.77 %
Folliculitis	528	0.50 %	7.77 %	0.00 %	0.00 %	0.19 %	0.95 %	7.20 %
Impetigo	310	0.30 %	8.06 %	0.00 %	0.65 %	0.32 %	0.97 %	6.45 %
Furuncle	363	0.35 %	13.77 %	0.28 %	0.83 %	1.93 %	3.31 %	10.19 %
Mastitis	1,533	1.46 %	6.07 %	0.13 %	0.20 %	0.33 %	0.33 %	5.28 %
Surgical Site Infection	20,849	19.86 %	23.86 %	0.12 %	0.59 %	0.77 %	6.05 %	18.78 %
Decubitus Ulcer	10,140	9.66 %	35.12 %	0.07 %	1.16 %	9.67 %	18.19 %	18.29 %
Device or Graft	13,204	12.58 %	44.21 %	0.07 %	0.23 %	0.73 %	8.58 %	37.73 %
Non-healing Surgical Wound	1,861	1.77 %	18.27 %	0.05 %	1.18 %	3.44 %	6.34 %	10.21 %
Other	2,517	2.40 %	17.40 %	0.24 %	1.03 %	2.54 %	7.47 %	9.81 %
All	104,972	100.01 %	23.28 %	0.61 %	1.07 %	2.29 %	6.92 %	16.33 %

^a^Complications are not mutually exclusive

^b^ Any inpatient occurrence with an SSTI diagnosis or SSTI complication following the index date

^c^Abscess/cellulitis category includes erysipelas (1,035 in ambulatory settings and 78 in inpatient settings)

For inpatient-treated SSTIs, several categories of SSTIs had higher proportion of diagnosis than compared to their proportion in the ambulatory-treated group. These categories included surgical site infections (19.86 %, 95 % CI: 19.81–19.91 %)), SSTIs associated with devices or grafts (12.58 %, 95 % CI: 12.54–12.62 %)) and decubitus ulcers (9.66 %, 95 % CI: 9.62–9.70 %), *p* < 0.0001 for all comparisons) (Table 3). Overall, rates of complications of SSTIs including lymphadenitis, myositis, necrotizing fasciitis, gangrene, osteomyelitis, bacteremia, endocarditis, septicemia and sepsis, were 1.16 % (95 % CI: 1.15–1.17 %) in the outpatient-treated SSTIs and 23.28 % (95 % CI: 23.02–23.54 %) in the inpatient-treated SSTIs (Table 3). Additional complications of SSTIs and proportion patients of re-hospitalized stratified by initial SSTI category and treatment location are detailed in Table 3.

**Fig. 1:**
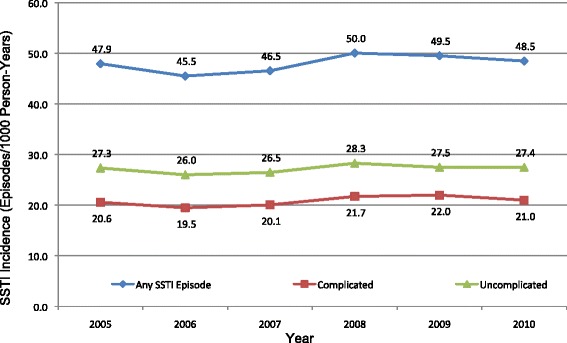
Incidence of skin and soft tissue infections (SSTIs) by severity from 2005 through 2010. Note: 95 % confidence interval bars are not included, as the confidence intervals very narrow and would not be visible in the figure

Temporal trends in SSTIs are summarized in Fig. 1. Despite a slight decrease from 2005 to 2007, SSTI incidence remained relatively unchanged from 2005 to 2010, from 47.94 (95 % CI: 47.79–48.09) to 48.46 (95 % CI: 48.31–48.61) SSTIs per 1,000 PY. In this time period, the incidence of uncomplicated SSTIs also remained relatively stable from 27.34 (95 % CI: 27.23–27.46) to 27.45 (95 % CI: 27.33–27.57) SSTIs per 1,000 PY, as did complicated infections 20.57 (95 % CI: 20.47–20.67) to 20.99, (95 % CI: 20.88–21.09) SSTIs/1,000 PY. Although all trends were statistically significant (*p* < 0.0001 for all comparisons), the magnitude of change was relatively small.

**Fig. 2:**
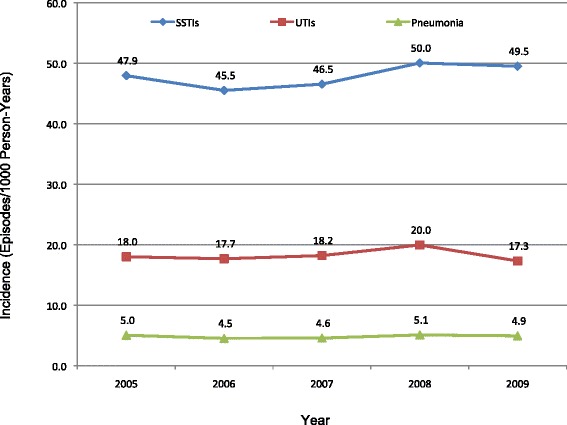
Incidence of skin and soft tissue infections (SSTIs), pneumonia and urinary tract infections (UTIs) from 2005 through 2010. Abbreviations: SSTI = Skin and Soft Tissue infection; UTI = Urinary Tract Infection. Note: 95 % confidence interval bars are not included, as the confidence intervals very narrow and would not be visible in the figure

In our comparative examination of other common infectious diseases from 2005 to 09, we found an incidence of UTIs, ranging from 17.31 to 19.95 infections per 1,000 PY during the study period, and incidence of pneumonia ranging from 4.59 to 5.03 per 1,000 PY during the study period (Fig. 2).

## Discussion

We performed an examination of both ambulatory- and inpatient-treated SSTIs as part of a single investigation in a large cohort of patients. We analyzed data from an administrative database and identified over 2.3 million SSTIs between 2005 and 2010. We found that the incidence of SSTIs is substantial and approximately twice of that of UTIs and tenfold of that of pneumonia. In our study period, on average, over 4 SSTIs occurred per 100 persons aged 0–64 years old annually. We also found that SSTIs were about twenty times more likely to be ambulatory-treated than inpatient-treated. Finally, our findings suggest that the rapid increase in SSTI incidence observed earlier during this Century [1, 2, 5] has leveled off, and SSTI incidence has been relatively stable across our study period.

We used ICD-9 codes to identify SSTI occurrences, which is a research identification method similar (or identical) to previous SSTI investigations [1, 2, 5]. While diagnosing SSTIs using ICD-9 coding has limitations (i.e., potential misclassification, coding errors), we observed that our SSTI incidence was comparable to other studies that used different administrative claims databases. Hersch et al. estimated outpatient infections in the U.S. and found that the visit rate among patients aged 18–44 years increased from 13.1 visits per 1,000 population in 1997 to 27.1 in 2005 [1]. Their 2005 estimates compare to our findings of 42.9 ambulatory-treated infections per 1,000 population per year between 2005 and 2010 in this age group. However, they used data from the National Ambulatory Medical Care Survey, which did not sample non-physician clinician visits in office-based practices, which may in part explain their lower incidence [16]. Edelsberg et al. found that an estimated 289,715 admissions occurred for SSTIs among patients aged 45–64 years in 2004 [5]. Our estimate of SSTIs with inpatient-treated of 3.9 per 1,000 PY in the 45–64 age category represents about 277,000 episodes during 2004, based on U.S. census population estimates for this year [17]. These results suggest that our findings of SSTI incidence are valid in comparison to other investigations employing claim-based methodologies. Finally, our SSTI incidence over the study period of 47.9 SSTIs/1000 person years is also similar to that the 49.6 SSTIs/1000 person year rate found in a population of Kaiser Permanente Northern California patients from 2009 to 2011 when analyzing electronic medical records and administrative databases [18].

Our study is novel as it compares SSTI incidence to other common infectious diseases. The reasons for these comparisons were twofold. First, if a trend was noted SSTI incidence, we wanted to ensure that these represented true incidence changes rather than secular changes in the HIRD population or other methodological factors. However, SSTI incidence changes observed were relatively modest and likely represented year to year fluctuations rather than true trends observed between the late 1990s and 2005 in other populations [1, 2, 5]. The second reason for comparing SSTI trends to other common infectious diseases is descriptive. We chose pneumonia and UTIs given they are relatively common infections seen in both inpatient and ambulatory settings and affect both children and adults. We found an SSTI incidence approximately twice of that of UTIs and 10 fold of that of pneumonia. The UTI incidence of 17.3–20.0 UTIs per 1,000 PY is comparable to findings from outpatients <65 years in Veteran Administration (VA) clinics in 2001. Among females, our UTI incidence was 31.43 UTIs/1,000 PY (data not shown), which is comparable to the estimated 55.1–60.6 UTIs per 1,000 PY in VA clinics [11]. Among males, UTI incidence was 5.23 UTIs/1,000 PY (data not shown), which is comparable to the estimated 16.7–22.7 UTIs per 1,000 PY men [12]. Our lower rate may be related to the fact that the VA does not care for children, in which the incidence of UTI is very low [11, 12], and the VA population may not be representative of insured adults. When these facts are considered, the rates may be more comparable. Our pneumonia incidence we found was comparable to ICD-9 code data from the very large MarketScan Commercial database, in which the pneumonia incidence of 4.89 episodes per 1,000 PY among those aged 18–64 [13] is comparable to our incidence of 4.10–5.03 per 1,000 PY.

Our study has several strengths. First, we studied trends in a demographically diverse population that included a very large group of persons with and without significant co-morbidities. Second, the time period of our investigation was novel in that it examined SSTI incidence in the period after a rapid rise in SSTIs that occurred in the first 5 years of the 21^st^ Century [1, 2]. Third, we used databases that included both inpatient and outpatient treated SSTIs. Previous large studies in the U.S. captured skin infections only in ambulatory settings, and used sample sizes that were a thousand-fold smaller than the present research to create estimates of SSTI [1, 19]. SSTI investigations in hospitalized patients that used HCUP data utilized a stratified random sample of only inpatient stays for SSTI [5, 6]. Unlike these previous efforts, our investigation directly measured SSTI rates rather than analyzing a subset of cases or a convenience sample.

There are limitations to our investigation. First, due to the nature of the commercially insured data used, we did not include persons over 65, the majority of them who utilize a Medicare or fee-for-service reimbursement model instead of commercial insurance plans. If we were able to quantify SSTI incidence in older Americans, it is likely the rates of infection would be substantially higher in this age group compared to younger persons. Older Americans typically have a higher prevalence of co-morbidities associated with increased risk for SSTI [20] and they have a higher risk of many other infections probably due co-morbidities and declines in host defenses [21, 22]. Second, we did not have microbiologic correlations for each SSTI. Thus we could not assess if the relationship between CA-MRSA trends and SSTI incidence in our cohort. Nevertheless, other investigations have shown that CA-MRSA appears to be the primary cause of increasing community-associated SSTI incidence in the beginning around 2000 [23, 24]. Third, although we were able to categorize diagnoses of skin infection by clinical type, the accuracy of these designations is unclear as diagnoses were derived from billing codes that may or may not have reflected accurately the pathology of the actual infection seen by clinician. Fourth, our designation of uncomplicated and complicated may not necessarily be consistent with definitions used by others [14, 15]. Regardless, there is no standardized definition distinguishing complicated from uncomplicated infection and, when developed, such distinctions may only be useful for clinical trials in which clinicians are prospectively determining definitions based on clinical presentation [25]. Finally, our study population is selected from all the U.S. regions and some regions are more represented than others. However, overall the HIRD population is geographically diverse and covers most regions of the U.S.

## Conclusion

In summary, we found that SSTI incidence between 2005 and 2010 has remained relatively constant, suggesting that with previously observed increases in SSTI incidence have now been stabilized. The SSTI incidence remained relatively steady among both ambulatory-treated and inpatient-treated infections as well as among both complicated and uncomplicated infections. Between 2005 and 2010, approximately 4.8 SSTIs requiring medical attention annually occurred per 100 person years among those aged 64 years and younger. Given the high incidence of SSTIs, interventions to prevent SSTIs would have great potential to reduce disease morbidity and health care utilization.

## Abbreviations

CA-MRSA: Community-associated methicillin-resistant *Staphylococcus aureus*
HCUP: Healthcare Cost and Utilization Project
HIPAA: Health Insurance Portability and Accountability Act
HIRD: HealthCore Integrated Research Database
ICD-9: International Statistical Classification of Diseases and Related Health Problems, Ninth Revision
PHI: Protected health information
PY: Person-years
SSTI: Skin and soft tissue infection
US: United States
UTI: Urinary tract infections
VA: Veteran Administration

## References

[1] Hersh AL, Chambers HF, Maselli JH, Gonzales R (2008). National trends in ambulatory visits and antibiotic prescribing for skin and soft-tissue infections. Arch Intern Med.

[2] Pallin DJ, Egan DJ, Pelletier AJ, Espinola JA, Hooper DC, Camargo CA (2008). Increased US emergency department visits for skin and soft tissue infections, and changes in antibiotic choices, during the emergence of community-associated methicillin-resistant *Staphylococcus aureus*. Ann Emerg Med.

[3] Carratala J, Roson B, Fernandez-Sabe N, Shaw E, del Rio O, Rivera A (2003). Factors associated with complications and mortality in adult patients hospitalized for infectious cellulitis. Eur J Clin Microbiol Infect Dis.

[4] Lipsky BA, Kollef MH, Miller LG, Sun X, Johannes RS, Tabak YP (2010). Predicting bacteremia among patients hospitalized for skin and skin-structure infections: derivation and validation of a risk score. Infect Control Hosp Epidemiol.

[5] Edelsberg J, Taneja C, Zervos M, Haque N, Moore C, Reyes K (2009). Trends in US hospital admissions for skin and soft tissue infections. Emerg Infect Dis.

[6] Levit K, Wier L, Stranges E, Ryan K, Elixhauser A (2009). HCUP facts and figures: statistics on hospital-based care in the United States, 2007.

[7] Kessler RC, Coulouvrat C, Hajak G, Lakoma MD, Roth T, Sampson N (2010). Reliability and validity of the brief insomnia questionnaire in the America insomnia survey. Sleep.

[8] Kim SY, Schneeweiss S, Liu J, Daniel GW, Chang G, Garneau K (2010). Risk of osteoporotic fracture in a large population-based cohort study of patients with rheumatoid arthritis. Arthritis Res Ther.

[9] Lee TA, Chang C-L, Stephenson JJ, Sajjan SG, Maiese EM, Everett S (2010). Impact of asthma controller medications on medical and economic resource utilization in adult asthma patients. Curr Med Res Opin.

[10] Phillips S, Delorenze GN, Quesenberry C, Willey VJ, Niemcryk SJ, Wells K (2010). Epidemiologic study of ariprazole use and the incidence of suicide events. Pharmacoepidemiol Drug Saf.

[11] Griebling TL, Litwin MS, Saigal CS (2007). Urinary tract infection in women. Urologic diseases in America.

[12] Griebling TL, Litwin MS, Saigal CS (2007). Urinary tract infection in men. Urologic diseases in America.

[13] Bonafede MM, Suaya JA, Wilson KL, Mannino DM, Polsky D (2012). Incidence and cost of CAP in a large working-age population. Am J Manag Care.

[14] Food and Drug Administration (1998). Guidance for industry. Uncomplicated and complicated skin and skin structure infections-developing antimicrobial drugs for treatment. Draft guidelines 1998 [monograph on the internet].

[15] Stevens DL, Bisno AL, Chambers HF, Everett ED, Dellinger P, Goldstein EJ (2005). Practice guidelines for the diagnosis and management of skin and soft-tissue infections. Clin Infect Dis.

[16] Hing E, Hooker RS, Ashman JJ (2011). Primary health care in community health centers and comparison with office-based practice. J Community Health.

[17] U.S.Census Bureau, Population Division. National Population Projections Released 2008 (Based on Census 2000) [monograph on the Internet]. Washington, U.S.Census Bureau 2008 [cited 2008 Jan]. 2008. Available from: https://www.census.gov/population/projections/data/national/2008/summarytables.html.

[18] Ray GT, Suaya JA, Baxter R (2013). Incidence, microbiology, and patient characteristics of skin and soft-tissue infections in a U.S. population: a retrospective population-based study. BMC Infect Dis.

[19] McCaig LF, McDonald LC, Mandal S, Jernigan DB (2006). *Staphylococcus aureus*-associated skin and soft tissue infections in ambulatory care. Emerg Infect Dis.

[20] Capitano B, Leshem OA, Nightingale CH, Nicolau DP (2003). Cost effect of managing methicillin-resistant *Staphylococcus aureus* in a long-term care facility. J Am Geriatr Soc.

[21] Chong C, Street PR (2008). Pneumonia in the elderly: a review of the epidemiology, pathogenesis, microbiology, and clinical features. South Med J.

[22] Wenger JD, Hightower AW, Facklam RR, Gaventa S, Broome CV (1990). Bacterial meningitis in the United States 1986: report of a multistate surveillance study. The bacterial meningitis study group. J Infect Dis.

[23] King MD, Humphrey BJ, Wang YF, Kourbatova EV, Ray SM, Blumberg HM (2006). Emergence of community-acquired methicillin-resistant *Staphylococcus aureus* USA 300 clone as the predominant cause of skin and soft-tissue infections. Ann Intern Med.

[24] Moran GJ, Krishnadasan A, Gorwitz RJ, Fosheim GE, McDougal LK, Carey RB (2006). Methicillin-resistant S. aureus infections among patients in the emergency department. New Engl J Med.

[25] O’Sullivan CE, Baker MG (2010). Proposed epidemiological case definition for serious skin infection in children. J Paediatr Child Health.

